# The State’s obligation to regulate and monitor private health care facilities: the Alyne da Silva Pimentel and the Dzebniauri cases

**DOI:** 10.1186/s40985-017-0063-6

**Published:** 2017-08-02

**Authors:** Ximena Andión Ibañez, Tamar Dekanosidze

**Affiliations:** 1 LLM International Human Rights Law, University of Essex, Colchester, UK; 2 Georgian Young Lawyers’ Association, 24 D. Kipiani Street, 0154 Tbilisi, Georgia; 3 Instituto de Liderazgo Simone de Beauvoir, Malaga 17, Depto. 6, Col. Insurgentes Mixcoac, C.P, 03920 Ciudad de México, Mexico

**Keywords:** Private health care, Patient rights, State responsibility, Human rights in patient care

## Abstract

The Human Rights in Patient Care framework embraces general human rights principles applicable to both patients and health care providers in the delivery of health care. Under this framework, states have a duty to ensure patient and provider rights in both public and private health care settings. The paper examines the recent decisions in *Alyne Da Silva Pimentel v. Brazil* of the Committee on the Elimination of Discrimination against Women and *Dzebniauri v. Georgia* of the European Court of Human Rights and places these decisions within the wider debate on the extent to which states have human rights obligations in private settings. Drawing on these decisions, the paper demonstrates that this duty can be complied with by establishing appropriate laws and regulations for private entities, monitoring and enforcement of the standards, and performance of these bodies and professionals through investigation and accountability procedures.

## Background

The concept of “human rights in patient care” embraces a set of human rights principles applicable in the context of health care provision in both public and private health facilities. It is designed to look comprehensively to the human rights compromised in patient care and to look both at patients and providers. It focuses on systemic issues, placing particular attention on marginalized groups, and it includes both patients and health care providers [1].

Given the global trend to privatize health care settings, one of the questions is to what extent the states are internationally responsible regarding the acts committed in private settings and what specific obligations do states have towards these private institutions. Under international human rights law, states can be held internationally responsible for the abuses committed in private health care settings. However, the extent of the state obligation towards the acts of the private individuals could vary, which is increasingly the subject of interpretation by international, regional, and even national human rights bodies.

This article tries to answer this question by looking from the human rights in patient care framework to the general obligations that states have to ensure the respect, protection, and fulfillment of the rights of patients in private settings and illustrating the evolution of the standards in this respect with the recent decisions of the European Court of Human Rights (ECHR) *in Dzebniauri v. Georgia* [2] and the Committee on the Elimination of Discrimination against Women (CEDAW Committee) in *Alyne Da Silva Pimentel v. Brazil* [3].

The paper first examines the debate in international human rights law on state accountability regarding acts of private actors in the field of health care and situates the issue in the human rights in patient care framework. After this, the paper analyses the decisions in the *Dzebniauri* and *Da Silva Pimentel* cases placing them within the discussion on state responsibility in private settings. The paper concludes that the decisions set important precedents to interpret the state obligations for the acts of private entities and the state obligation to provide the remedy for the abuses committed in private settings.

## State human rights obligations for the acts of non-state/private actors

There is a growing set of standards, at the international and regional levels, of the state human rights obligations regarding non-state actors, as well as the human rights responsibilities that private actors have under international human rights law. There are also many discussions as to how the responsibilities of private actors, particularly corporations, can be expanded (for a more in depth discussion see [4–8]).

International human rights law establishes that states have the primary obligation to respect, protect, and fulfill human rights [9] in the context of, inter alia, state-owned or private enterprises/institutions [10]. As related to the right to health, the obligation to *respect* requires that the states shall not interfere, directly or indirectly, with the enjoyment of this right. Under the obligation to *protect,* the states are required to take measures that prevent third parties from interfering with the guarantees under the right to health. Finally, the obligation to *fulfill* provides that the states shall adopt appropriate legislative, administrative, budgetary, judicial, promotional, and other measures for the full realization of the right to health [11].

There is no requirement of a specific economic or political system, under international human rights, for effective realization of economic and social rights, including the right to health. The Committee on Economic, Social and Cultural Rights recognizes that these rights “are susceptible of realization within the context of a wide variety of economic and political systems” [12, 13]. This implies that a certain division between the state and the marketplace is not required, and private actors can also have a role in the realization of health rights. In this context, the question becomes how to define their obligations under international human rights law and how to ensure their accountability.

In relation to private health care settings, under the obligation to respect, states should also ensure that there are laws and regulations in place that ensure that private health care services are affordable, accessible, acceptable, and of good quality [12, 13]. The CEDAW Committee has established that states have the obligation to “ensure that public and private health-care providers meet their duties to respect women’s rights to have access to health care” [14].

The obligation to protect implies that the state needs to ensure that those laws and regulations are appropriately implemented and that violations of human rights that occur in private health settings are appropriately investigated, redressed, and prevented [14]. Therefore, the obligation to protect is applicable when it comes to human rights abuses committed in private health care settings.

Responsibility to protect is engaged in the practice of the ECHR, which has recognized that positive obligations under Article 2 (right to life) of the European Convention on Human Rights (*hereinafter* the European Convention) “require States to make regulations compelling hospitals … to adopt appropriate measures for the protection of their patients’ lives” and “an effective independent judicial system to be set up so that the cause of death of patients in the care of the medical profession, whether in the public or the private sector, can be determined and those responsible made accountable …” [15]. The CEDAW Committee has established that the obligation to protect includes “rights relating to women’s health requires states parties, their agents and officials to take action to prevent and impose sanctions for violations of rights by private persons and organizations” including by ensuring an effective judicial system [14].

The Inter-American Court of Human Rights has also established that “the duty of the states to regulate and supervise the institutions which provide health care services, as a necessary measure aimed at the due protection of the life and integrity of the individuals under their jurisdiction, includes both public and private institutions which provide public health care services, as well as those institutions which provide only private health care” [16].

## The scope of the state responsibility for the abuses committed in private health care settings under *Da Silva Pimentel v. Brazil* and *Dzebniauri v. Georgia*

The 2014 ECHR decision of *Dzebniauri v. Giorgia* and the 2011 CEDAW Committee decision in *Da Silva Pimentel v. Brazil* are some of the recent decisions addressing the issue of state responsibility for violations committed in private health care settings. These decisions contribute to the development of international and regional jurisprudence and pave the way for more standard setting on this matter.

*Dzebniauri v. Georgia* is the first decision against Georgia, delivered by a regional body, addressing the obligation of the state to ensure human rights of a patient in a private civilian hospital. On the other hand, *Da Silva Pimentel v. Brazil* is the first case decided by an UN Human Rights Treaty Body holding a government responsible for a preventable maternal death—something that has long been ignored as a human rights issue.

### Dzebniauri v. Georgia

After the fall of the Soviet Union in 1991, Georgia’s health care system started to change from its universal accessibility model to vertical targeted health care programs. In 2005–2006, the state embarked on carrying out targeted social programs in cooperation with private insurance companies, with increasing privatization of state health care facilities. In 2013, the state started to implement the Universal Health Care Program to ensure the universal accessibility of health care services through providing insurance to all its citizens [17]. Human rights violations in health care settings remain prevalent, starting from the violations of the right to life to the range of other abuses of human rights in patient care [18].

On 9 April 2005, Giorgi Dzebniauri died in a private hospital in Tbilisi, Georgia as a result of the surgery of his inflamed gallbladder. Three months after the death a criminal investigation started into the alleged medical error causing Mr. Dzebniauri’s death. During the investigation different agencies conducted three forensic medical examinations, two of which confirmed the errors in the acts of the doctor. The same errors were also reiterated in the statement of the doctor’s assistant.

Despite the above evidence, for years the criminal investigation did not move any further, leading to the expiration of the statute of limitations for criminal negligence, after which charges could no longer be brought against the responsible doctor. Therefore, relying on Article 2 (right to life) of the European Convention, the mother of the deceased filed an application at the ECHR on 7 October 2011. She complained that the state had violated its positive obligations, as (a) the state had failed to ensure a proper and risk-safe functioning of the relevant private civilian hospital; and (b) no meaningful investigation was carried out into the medical errors, which had caused the death of the her son.

After the exchange of several submissions with the applicant on the substance of the case and attempts of friendly settlement, the Government of Georgia made a unilateral declaration and acknowledged the violation of its positive obligations under Article 2. In particular, the Government had failed to properly inspect the private medical institution for its compliance with the license conditions; and there were “certain deficiencies” in the treatment provided to the patient. In addition, the Government acknowledged that there were certain deficiencies in the investigation of the death of the applicant’s son, violating the procedural obligation under Article 2 of the Convention. To remedy these violations, the Government offered the applicant the compensation to cover any pecuniary or non-pecuniary damages and costs and expenses. However, the applicant vehemently refused any compensation offers, stating that no money could remedy the suffering resulting from the death of her son [2].

Since the Government unequivocally acknowledged the violation of its positive obligations under Article 2, the Court did not decide the case on the merits. The Court found that the declaration of the Government was based on the respect of human rights, found no other reasons for a continued examination of the application and struck the case out of the list [2].

### Alyne Da Silva Pimentel v. Brazil

Maternal mortality in Brazil is particularly prevalent among low income, Afro-Brazilian and indigenous women [19]. Since 1988 Brazil has developed a Unified Health System (SUS) based on the decentralization of management and provision of services, the strengthening of primary health care services and the promotion of community participation. Although the system promotes universal access and equity, it still faces great challenges in achieving this goal, including the inequity of government funding for different regions and the participation of the private sector [20].

The *Alyne Da Silva Pimentel v. Brazil* case concerns an Afro-Brazilian woman resident in one of the poorest districts of Rio de Janeiro who died during pregnancy because of the lack of access to adequate and quality health care services. When Alyne was 6 months pregnant, she went to a private health clinic with symptoms of a high-risk pregnancy and she was sent back home without any proper diagnosis. The symptoms worsened so she went back again to the clinic and, after being yelled at by the health personnel, she was finally admitted. When doctors conducted an ultrasound, they could not find a fetal heartbeat so they had to induce delivery, and in the process, they left a piece of placenta inside that caused an infection. Her condition worsened and she needed to be transferred to a secondary health facility to get a blood transfusion. She had to wait more than 8 h before being transferred to a tertiary health facility where she was left without proper attention and finally died in a hallway of the hospital. Alyne’s death was entirely preventable.

Alyne’s family presented a civil claim against the State of Rio de Janeiro demanding material and moral damages for her preventable death. After 4 years without any response from Brazil’s judicial system, the Center for Reproductive Rights and Advocaci presented the case before the CEDAW Committee arguing Brazil’s breach of its obligations to ensure non-discrimination in access to quality health care services during pregnancy and childbirth and protect the right to life and the right to access justice.

In 2011 the CEDAW Committee issued its decision on the case finding the State of Brazil responsible for violations of article 2(c) (access to justice); article 2(e) (the state’s obligation to regulate private health care facilities), in conjunction with article 1 (non-discrimination) and article 12 (right to health) [3]. In this decision, the CEDAW Committee established that Alyne’s family needs to be adequately redressed and it also recommended a series of measures to improve access and quality of maternal health care services in Brazil as well as mechanisms for monitoring and accountability.

After this decision was issued, in 2013, the judicial system in Brazil also decided over the claim presented and awarded moral damages and a pension for Alyne’s daughter until she is 18. Nevertheless, it did not find the state directly responsible for the violations in the private health care clinic [19].

### Responsibility of the state for the acts of private medical institutions in Da Silva Pimentel and Dzebniaui cases

Although the factual circumstances and context of *Dzebniauri* and *Da Silva Pimentel* cases differ, international and regional bodies in both cases raise similar issues of state responsibility in relation to the acts committed in private health care settings. While in the *Dzebniuari* case, the Government of Georgia acknowledged a violation in connection with the human rights abuse committed by the private entity, in *Da Silva Pimentel* the CEDAW Committee decided the case on the merits and determined the scope of the state responsibility.

The main legal issues the two cases raise is whether the states were responsible since the abuses, negligence and omissions were committed in private health care facilities. Brazil argued that the state was not responsible since it was a private facility, while Georgia decided to acknowledge its responsibility before the ECHR could elaborate on it. Respectively, both the ECHR and the CEDAW Committee reaffirmed that acts committed in private health care settings gave rise to state responsibility.

One of the starting points in the *Da Silva Pimentel* case was the acknowledgment that health is a public good and a right and that even when the states transfer the service provision to private corporations they still have primary responsibility for the respect, protection and fulfillment of human rights in patient care contexts. The CEDAW Committee established, taking into account the protection of the right to health under Brazil’s Constitution, that “the state is directly responsible for the action of private institutions when it outsources its medical services and that, furthermore, the state always maintains the duty to regulate and monitor private health-care institutions” [3].

After asserting that the ultimate responsibility is the state’s, in both decisions there is a profound analysis as to how the states failed to comply with the responsibility to ensure the respect and protection of the human rights in care of patients in private health care facilities. Both decisions emphasize the lack of adequate regulations and oversight mechanisms to ensure the provision of quality health care services. In the *Dzebniauri* case, the ECHR accepted the Government’s acknowledgement as follows*:*

“Bearing in mind shortcomings acknowledged with regard to the inspection of the respective medical establishment concerning the compliance of medical licence conditions prior to the incident of Mr. Dzebniauri’s death; Acknowledging certain deficiencies identified in the course of the medical treatment dispensed to the applicant’s son in the private medical establishment known under the name of ‘Lechkombinati’.” [2]

Therefore, the ECHR accepted the acknowledgement of the Government of the specific duties to protect the right to life of patients in private medical establishments. In particular, this duty entails putting in place a system of effective inspection of private hospitals, including checking compliance with the license conditions.

In the *Da Silva Pimentel* case, the CEDAW Committee acknowledged that “the State party has a due diligence obligation to take measures to ensure that the activities of private actors in regard to health policies and practices are appropriate” (for more on this see [21, 22]).^1^ In addition, it determined that the state had failed to ensure effective judicial action and protection given the delay in the judicial proceedings at the national level [3] (For a robust analysis of the implications of the *Da Silva Pimentel* decision see [23]).

Therefore, both decisions acknowledged that the lack of adequate and quality health care services provided in a private health care facility can give rise to the state’s responsibility for not protecting the right to life of patients.

This obligation to ensure the provision of quality services is enhanced in the case of Alyne since she was an Afro-Brazilian woman and there was a specific obligation to ensure non-discrimination and equal access to health care services. In the *Da Silva Pimentel* case the CEDAW Committee developed a very strong substantive equality analysis of facts of the case and asserted that “the lack of appropriate maternal health services has a differential impact on the right to life of women” [3]. It also reaffirmed that gender discrimination in this particular case was connected with discrimination based on race and income, all of which conditioned Alyne’s access to quality health care services. The Committee assessed Brazil’s efforts to combat maternal mortality and established that the core obligations of the states to respect, protect and fulfill rights includes that the policies of the state are “action-and result-oriented as well as adequately funded” [3].

Furthermore, in both cases the international and regional bodies paid special attention to the fact that there was a failure to provide adequate remedies and redress for the victims at the national level. This was another violation to the state obligation to protect the rights of patients in health care. The ECHR in the *Dzebniauri* case reaffirmed that the state’s duty was ensuring that private hospitals provide the type of medical treatment that respects the right to life of patients; and if a patient dies as a result of medical malpractice, the Government has the duty to have an effective and independent judicial system, to determine the cause of death and bring those responsible (relevant medical personnel or medical institution) to account [15].

In the *Da Silva Pimentel* case, the CEDAW Committee goes beyond this reasoning, reaffirming the state’s obligations to provide effective judicial remedies and redress for violations of reproductive rights of women. The Committee established that health care providers should be held accountable for their actions and omissions that violated the right to health, non-discrimination and life [3].

Although the CEDAW decision is groundbreaking, there are certain points that were not addressed in the depth that was expected. For instance, the CEDAW Committee neglected important structural issues in the health care system in terms of discrimination based on gender, race and socio-economic status that impeded access to appropriate health care services for Afro-descendant women in Brazil. This is unfortunate considering the relevance it has, as it is laid out in the human rights in patient care framework [1].

As to the *Dzebniauri* case, despite the unequivocal acknowledgment of the violations by the Government, the decision does not include the obligation to undertake general measures to ensure the compliance with the decision and to prevent further violations of the right to life in private health care settings. Even though the decision is binding, the lack of the obligation to undertake general measures makes it problematic to enforce in practice. Given that by the time of the decision the statute of limitation had expired to bring the doctor in charge to account, and the applicant refused to accept any compensation for damages, without wider lobbying more advocacy is needed to push the Government to take measures to ensure human rights of patients in private health care institutions.

## Conclusions

Decisions in the *Da Silva Pimentel* and *Dzebniauri* cases affirm the already evolved international and regional standards that states are responsible for protecting the rights holders against human rights abuses in private health care settings and for remedying state violations connected with such abuses. The decisions pave the way for more standard setting on this matter by providing specificity on how this duty can be complied with, including by establishing appropriate laws and regulations for private entities and monitoring and enforcement of the set standards and performance of these bodies and professionals through investigation and accountability procedures.

The cases are a practical demonstration of how the “duty to protect” can be interpreted by international and regional human rights bodies in relation to the lack of adequate health care services when provided in private settings. This is particularly relevant in the current stage of the development of human rights in patient care, since it reaffirms that human rights are applicable in the delivery of health care services in all contexts, and that states can be held accountable for structural abuses even if they happen in private health care settings.

## Abbreviations

CEDAW: Committee on the Elimination of Discrimination against Women
ECHR: European Court of Human Rights

## Footnotes

1 The due diligence standard establishes that States have to take all measures possible to prevent, investigate and punish human rights violations. This is an ad hoc standard that needs to be assess in specific cases to determine whether the State acted with due diligence. The due diligence comes from the jurisprudence of the Inter-American Court of Human Rights in the case Velásquez Rodríguez v. Honduras, which deals with the State obligation to properly investigate and punish the disappearance of a person [21]. The standard has been largely used to assess States’ responsibility for gender-based violence. For more on this see: [22].
